# Delayed diagnosis of cystic fibrosis: clinical burden and missed opportunities in a cohort from Rio de Janeiro, Brazil

**DOI:** 10.36416/1806-3756/e20260136

**Published:** 2026-09-14

**Authors:** Renata Wrobel Folescu Cohen, Tania Wrobel Folescu, Leonardo Yllel Rocha, Monica Taulois, Patrícia Fernandes Barreto Machado Costa, Raphael Freitas Jaber de Oliveira

**Affiliations:** 1. Instituto Fernandes Figueira, Fundação Oswaldo Cruz - IFF/Fiocruz - Rio de Janeiro (RJ) Brasil.; 2. Universidade do Estado do Rio de Janeiro - UERJ - Rio de Janeiro (RJ) Brasil.

**Keywords:** Cystic fibrosis, Delayed diagnosis, Neonatal screening, Bronchiectasis, Brazil

## Abstract

**Objective:**

To describe clinical and epidemiological profile of patients with cystic fibrosis (CF) diagnosed outside neonatal screening programs at two referral centers in Rio de Janeiro, Brazil, and to assess the impact of diagnostic delay on disease severity at the time of diagnosis.

**Methods:**

This cross-sectional study included 54 patients with a confirmed diagnosis of CF evaluated at two CF referral centers in Rio de Janeiro between 2019 and 2023. Data collected included age at diagnosis, neonatal screening history, clinical manifestations at diagnosis, previous misdiagnoses, microbiological findings, pulmonary function, and nutritional status. Patients were stratified by age at diagnosis (< 5, 5-12, 13-17, and ≥ 18 years) and by pediatric versus adult diagnosis. Chi-square tests were used for categorical comparisons.

**Results:**

Among the 54 patients, 59.3% were male, and 98.1% were not identified by neonatal screening. Median age at diagnosis was 11 years (IQR: 4.5-23.5). Diagnosis during adulthood occurred in 31.5% of patients. Bronchiectasis was present in 88.2% of adults compared with 47.1% of pediatric cases (p = 0.0045). CF-related diabetes was exclusively observed in adults (41.2%; p < 0.001). Prior misdiagnosis occurred in 45 patients (83.3%), most commonly asthma (24.1%), tuberculosis (11.1%), or idiopathic bronchiectasis (7.4%). At diagnosis, the median FEV_1_ was 62% of the predicted value among patients with available spirometry. Bacterial airway infection was detected in 81.5% of cases.

**Conclusions:**

Late CF diagnosis in Rio de Janeiro occurred predominantly outside neonatal screening programs, with a median age at diagnosis of 11 years, reflecting substantial missed opportunities for early intervention. Diagnosis during adulthood is associated with higher rates of bronchiectasis and CF-related diabetes, highlighting the irreversible structural and metabolic burden associated with diagnostic delay.

## INTRODUCTION

Cystic fibrosis (CF) is the most common life-limiting autosomal recessive disorder, caused by pathogenic variants in the *CF transmembrane conductance regulator* (*CFTR*) gene on chromosome 7q31.2. The disease affects multiple organ systems, particularly the lungs, pancreas, liver, and reproductive tract, resulting in progressive morbidity and premature mortality if not identified and managed early.^1^ ^,^ ^2^ Since the introduction of universal newborn screening (NBS)-also known as neonatal screening-programs in high-income countries, the median age at diagnosis has decreased dramatically, enabling presymptomatic initiation of treatment and substantially improving long-term outcomes.^3^ ^,^ ^4^

In Brazil, the *Programa Nacional de Triagem Neonatal* (PNTN, nationwide newborn screening program) was expanded to include CF in 2001 through a two-tiered immunoreactive trypsinogen (IRT/IRT) protocol. Despite these regulatory advances, implementation has remained heterogeneous, and coverage continues to be suboptimal across different regions, particularly in the North and Northeast.^5^ ^,^ ^6^ In Rio de Janeiro, NBS for CF was implemented later than nationally recommended, becoming fully established around 2010. Programmatic gaps and historical inequities in access to confirmatory testing have contributed to delayed CF diagnosis for a substantial proportion of patients. Consequently, many individuals continue to be diagnosed only after the onset of clinical symptoms, often years after the disease has begun causing irreversible pulmonary and extrapulmonary damage.^7^

The natural history of CF is characterized by a progressive decline in lung function from early childhood, driven by recurrent bacterial infections, chronic neutrophilic airway inflammation, and structural remodeling that culminates in bronchiectasis.^8^ When diagnosis is delayed beyond the neonatal period, this pathogenic cycle proceeds unchecked. Epidemiological evidence from North American and European CF registries consistently demonstrates that patients diagnosed outside NBS programs have poorer lung function, higher rates of pulmonary exacerbations, greater nutritional impairment, and increased early mortality than those identified through NBS.^9^ ^,^ ^10^ In the era of highly effective CFTR modulators, the imperative to diagnose CF early has become even more compelling, as these therapies achieve their greatest benefit when initiated before irreversible structural damage has occurred.^11^

Despite the clinical and public health relevance of delayed CF diagnosis in Brazil, data from referral centers in the Southeast-particularly those serving socioeconomically diverse populations in Rio de Janeiro-remain scarce. Most published Brazilian studies have focused on genotype-phenotype correlations or lung transplantation outcomes and have not specifically characterized the burden of diagnostic delay and its associated clinical manifestations at the time of diagnosis. Describing this phenomenon at the local and regional levels is essential to generate evidence that can inform targeted screening policies, enhance diagnostic suspicion among clinicians, and support advocacy for programmatic improvements in NBS coverage.

In this context, the present study aimed to characterize the clinical and epidemiological profile of CF patients diagnosed outside NBS programs at two referral centers in Rio de Janeiro, quantify the interval between symptom onset and confirmed diagnosis, identify the most common misdiagnoses that preceded CF recognition, and evaluate the impact of age at diagnosis on indicators of clinical disease severity at the time of diagnosis.

## METHODS

This was a cross-sectional study conducted at two public referral centers for CF in the state of Rio de Janeiro, Brazil, between 2019 and 2023. These centers provide specialized care for patients referred through both the *Sistema Único de Saúde* (SUS, Brazilian Unified Health System) and the private healthcare sector across the metropolitan region of Rio de Janeiro.

Patients with a confirmed diagnosis of CF were included. The diagnosis of CF was confirmed by a positive sweat chloride test (≥ 60 mmol/L on two separate occasions, in accordance with current international guidelines) and/or the identification of two pathogenic *CFTR* variants by molecular genetic testing. Patients with an inconclusive diagnostic workup or incomplete medical records were excluded. All patients or legal guardians gave written informed consent. The study protocol was approved by the institutional ethics committee.

Data were extracted from medical records using a standardized data collection form. Variables collected included demographic characteristics (age and sex); NBS history; age at symptom onset and at confirmed CF diagnosis; prior diagnoses before CF confirmation; clinical manifestations present at diagnosis (respiratory symptoms, recurrent pneumonia, digital clubbing, hemoptysis, chronic rhinosinusitis, nasal polyposis, rectal prolapse, failure to thrive, and malnutrition); pulmonary function (FEV_1_ % predicted, when spirometry was available); chest CT findings (bronchiectasis, mucoid impaction, upper-lobe predominance); microbiological findings (bacterial, fungal, and mycobacterial cultures); pancreatic insufficiency; CF-related diabetes (CFRD); hepatobiliary disease; bone disease; and the specialty of the clinician who first suspected the diagnosis of CF.

Categorical variables are presented as absolute and relative frequencies, whereas continuous variables are presented as medians with interquartile ranges (IQR). Patients were stratified into two groups according to age at diagnosis into pediatric (< 18 years) and adult (≥ 18 years). Chi-square tests (without Yates’ correction) were used to compare categorical outcomes between the two groups. All statistical tests were two-sided, and a p value < 0.05 was considered statistically significant. For exploratory analyses, patients were further stratified into four age groups (< 5, 5-12, 13-17, and ≥ 18 years), and the prevalence of bronchiectasis was calculated for each stratum. Statistical analyses were performed using Python version 3.11 with the SciPy and Pandas libraries.

## RESULTS

A total of 54 patients with confirmed CF were included in the study. Of these, 32 (59.3%) were male. The median age at diagnosis was 11 years (IQR, 4.5-23.5; range 0-51 years), reflecting a wide age distribution, from the neonatal period to adulthood. Only 1 patient (1.9%) was identified through NBS; the remaining 53 patients (98.1%) were diagnosed following clinical investigation prompted by symptoms. Figure 1A shows the distribution of age at diagnosis. Diagnosis during adulthood (≥ 18 years) occurred in 17 patients (31.5%), whereas 37 (68.5%) were diagnosed during childhood or adolescence (Table 1).

**Table 1:** Demographic and clinical characteristics of patients with cystic fibrosis diagnosed outside neonatal screening (n = 54).

Variable	n	%
Male	32	59.3%
Female	22	40.7%
Neonatal screening, Yes	1	1.9%
Neonatal screening, No	53	98.1%
Age at diagnosis, years^*^	11	(4.5-23.5)
Age at diagnosis ≥ 18 years	17	31.5%
Age at diagnosis <18 years	37	68.5%
Symptom onset, Neonatal	10	18.9%
Symptom onset, Childhood	30	56.6%
Symptom onset, Adolescence	9	17.0%
Symptom onset, Adulthood	4	7.5%
Prior hospitalization before diagnosis	33	66.0%
Persistent respiratory symptoms	44	86.3%
Bronchiectasis at diagnosis	31	61.0%
Pancreatic exocrine insufficiency	30	60.0%
CF-related diabetes	7	13.5%
Malnutrition	21	42.0%
Positive bacterial culture	44	84.6%
Sweat chloride, mmol/L	89	66-98
FEV_1_, % predicted^a,*^	62	(49.5-78.0)
Prior misdiagnosis documented	45	83.3%

a n = 23. *Values expressed as median (IQR).

**Figure 1:**
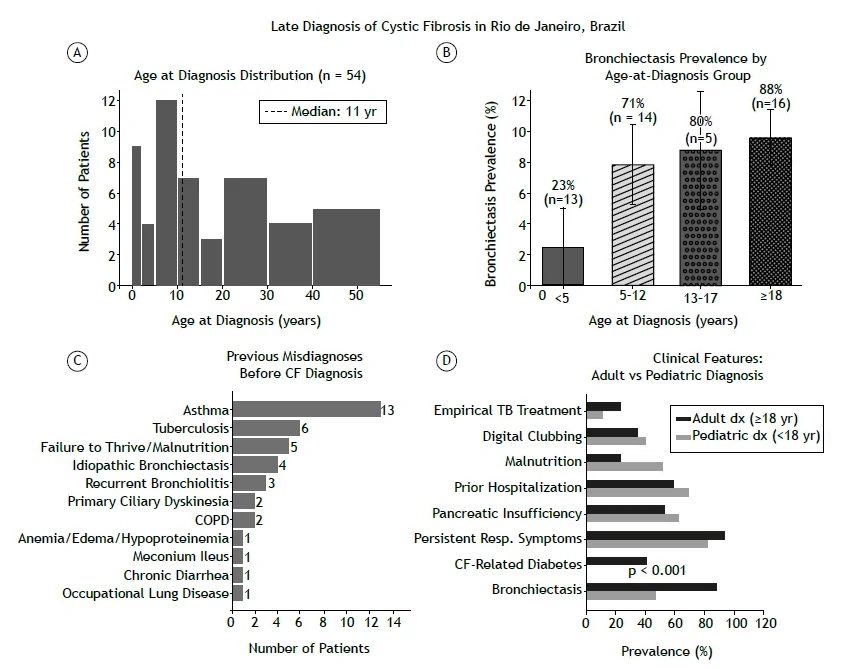
Clinical and epidemiological profile of patients with cystic fibrosis (CF) diagnosed outside neonatal screening in Rio de Janeiro, Brazil. In A, distribution of age at diagnosis (N = 54). In B, prevalence of bronchiectasis stratified by age-at-diagnosis group, with 95% confidence intervals. In C, most common prior misdiagnoses before diagnostic confirmation of CF. In D, prevalence of key clinical features at diagnosis comparing adult (≥ 18 years) and pediatric (<1 8 years) groups.

Symptom onset occurred during the neonatal period in 10 patients (18.9%), during childhood in 30 (56.6%), during adolescence in 9 (17.0%), and during adulthood in 4 (7.5%). Despite the early onset of symptoms in the majority of patients, a substantial diagnostic delay was observed, with most individuals receiving a confirmed diagnosis several years after symptom onset.

Prior misdiagnosis before CF confirmation was documented in 45 of 54 patients (83.3%; Figure 1C). The most common misdiagnoses were asthma (n = 13; 24.1%), tuberculosis (n = 6; 11.1%), failure to thrive/malnutrition (n = 5; 9.3%), idiopathic bronchiectasis (n = 4; 7.4%), recurrent bronchiolitis (n = 3; 5.6%), COPD (n = 2; 3.7%), and primary ciliary dyskinesia (n = 2; 3.7%). Five patients (9.3%) received empirical antituberculosis therapy before CF was considered. A pulmonologist was involved in the diagnostic pathway in 16 cases (45.7% of those with available data), and the specialty that first suspected CF was pulmonology in 11 cases (34.4%), pediatrics in 8 (25.0%), and gastroenterology, hepatology, or nutrition in 5 (15.6%).

Persistent respiratory symptoms were the most frequent clinical finding at diagnosis, present in 44 of 51 patients with available data (86.3%). Bacterial airway infection was documented in 44 of 52 patients (84.6%). The most frequently isolated pathogens were mucoid *Pseudomonas aeruginosa* (18 patients; 33.3%), non-mucoid *P. aeruginosa* (11 patients; 20.4%), and methicillin-sensitive *Staphylococcus aureus* (9 patients; 16.7%). Prior hospitalization before diagnosis was reported in 33 of 50 patients (66.0%). Pancreatic exocrine insufficiency was present in 30 of 50 patients (60.0%), malnutrition in 21 of 50 (42.0%), and CFRD in 7 of 52 (13.5%). The sweat chloride test was positive in all 53 patients who underwent testing, with a median chloride concentration of 89 mmol/L (IQR, 66-98 mmol/L). Spirometry at diagnosis was available for 23 patients and demonstrated a median FEV_1_ of 62% of the predicted value (IQR, 49.5-78.0%).

### Adult versus pediatric diagnosis: comparative analysis

Comparison between patients diagnosed during adulthood (≥ 18 years; n = 17) and those diagnosed during childhood or adolescence (<18 years; n = 37) revealed significant differences in disease burden at the time of diagnosis. Bronchiectasis was present in 88.2% of adults compared with 47.1% of pediatric-diagnosed patients (chi-square = 8.06; p = 0.0045) . CFRD was observed exclusively in adults (41.2% vs. 0.0%; chi-square = 16.65; p < 0.001). Malnutrition showed a trend toward a higher prevalence among pediatric patients (51.5% vs. 23.5%), although this difference did not reach statistical significance (p = 0.058). No statistically significant differences were observed between groups for pancreatic insufficiency, persistent respiratory symptoms, prior hospitalization, digital clubbing, or empirical antituberculosis treatment (Table 2).

**Table 2:** Comparison of clinical features at diagnosis: adult (≥ 18 years) vs. pediatric (< 18 years) groups.

Variable	Adult, ≥ 18 years (n = 17)	Pediatric, < 18 years (n = 37)	c²*	p
Bronchiectasis	88.2%	47.1%	8.06	0.0045
Cystic fibrosis-related diabetes	41.2%	0.0%	16.65	< 0.001
Persistent respiratory symptoms	94.1%	82.4%	1.32	0.250
Pancreatic exocrine insufficiency	52.9%	63.6%	0.53	0.465
Prior hospitalization	58.8%	69.7%	0.59	0.442
Malnutrition	23.5%	51.5%	3.61	0.058
Digital clubbing	35.3%	40.0%	0.11	0.744
Empirical tuberculosis treatment	23.5%	11.4%	1.29	0.257
Asthma misdiagnosis	17.6%	31.4%	1.10	0.293

*Chi-square test without Yates correction.

When patients were stratified into four age-at-diagnosis groups, the prevalence of bronchiectasis increased progressively with advancing age at diagnosis. Among patients diagnosed before age 5 years of age, bronchiectasis was present in 23.1% (3/13). This proportion increased to 71.4% (10/14) among those diagnosed between 5 and 12 years of age, 80.0% (4/5) among those diagnosed between 13 and 17 years, and 87.5% (14/16) among adults. These findings are illustrated in Figure 1B, which also depicts the distribution of age at diagnosis (Figure 1A), the spectrum of prior misdiagnoses (Figure 1C), and the comparison of clinical characteristics according to age at diagnosis (Figure 1D).

## DISCUSSION

The present study characterizes the clinical and epidemiological profile of CF patients who were not identified through NBS at two referral centers in Rio de Janeiro, Brazil. Our findings reveal three interrelated and alarming patterns: an overwhelming failure of NBS to identify affected individuals (98.1% of patients were undetected), a substantial diagnostic delay, with a median diagnosis age of 11 years, and a significantly progressive disease burden-most critically, bronchiectasis-that increased with advancing age at diagnosis.

The near-total absence of NBS among our cohort contrasts sharply with outcomes reported in countries with well-established CF NBS programs. In the United States, where universal CF NBS has been implemented since 2010, more than 96% of CF cases are detected in the neonatal period, with diagnosis occurring shortly after birth in screen-detected patients.^3^ In Australia and most Western European countries, NBS coverage consistently exceeds 90%, resulting in comparable improvements in early diagnosis rates.^4^ ^,^ ^9^ In Brazil, although the PNTN expanded CF coverage nationally over the past two decades, implementation of this program has remained profoundly heterogenous across regions. Data from the Brazilian CF Registry indicate that NBS accounts for fewer than 40% of new diagnoses nationwide, with marked regional disparities, exceeding 60% in the South but remaining below 20% in the North and Northeast.^5^ ^,^ ^6^ Our findings, with only 1.9% of patients identified through NBS, suggest that NBS for CF remains critically inadequate even in a highly urbanized southeastern state, likely reflecting both limitations in program implementation and socioeconomic barriers to follow-up after reactive initial test results.

The epidemiological consequences of this limited screening coverage are evident in the clinical profile of our patients at the time of diagnosis. Bronchiectasis-the hallmark of irreversible structural pulmonary injury in CF-was present in 61% of patients overall. More importantly, its prevalence increased progressively across age-at-diagnosis groups, from only 23% among children diagnosed before 5 years of age, to 71% in those diagnosed at 5-12 years, to 80% in the 13-17 age group, and to 87.5% in adults. This gradient powerfully demonstrates that each year of diagnostic delay represents an additional period of uncontrolled airway infection, inflammation, and structural lung damage. These findings are consistent with longitudinal analyses from the European CF Society Patient Registry and the North American CF Foundation Patient Registry, which have demonstrated that lung function impairment and structural lung damage significantly become more advanced in patients not identified through NBS.^3^ ^,^ ^10^

The higher prevalence of bronchiectasis in adults at diagnosis (88.2%; p = 0.0045) and the exclusive occurrence of CF-related diabetes (41.2% vs. 0%, p < 0.001) underscore the association between delayed diagnosis and accumulated disease burden. CF-related diabetes results from progressive pancreatic destruction and declining insulin secretion, processes that evolve over the years and correlate with disease duration and cumulative pulmonary inflammation.^12^ Its occurrence exclusively among adults in our cohort not only implies a longer disease course, but also a prolonged period during which the metabolic machinery of the exocrine and endocrine pancreas has deteriorated. Both bronchiectasis and CFRD are associated with accelerated lung function decline, more frequent exacerbations, reduced transplant-free survival, and poorer response to CFTR modulator therapy.^11^ ^,^ ^13^

The historical perception of CF as a predominantly pediatric disease also represents an important barrier. Increased survival and the recognition of milder forms have led to a growing number of diagnoses during adulthood; however, clinical suspicion in this group remains limited. Similarly, preserved fertility and established parenthood in some women may falsely reduce diagnostic suspicion, contributing to delayed recognition of the disease.^9^

The remarkably high frequency of prior misdiagnosis (83.3%) observed in our cohort highlights deficiencies that extend beyond the NBS program alone. Asthma was the most frequent erroneous diagnosis (24.1%), which is unsurprising given the overlapping phenotype of CF respiratory disease with atopic airways disease in children-both presenting with wheeze, recurrent respiratory infections, and airflow obstruction. Critically however, the CF-associated asthma phenotype fails to respond adequately to standard the bronchodilator and inhaled corticosteroid therapy, a “red flag” that should prompt consideration of alternative diagnoses, including CF.^14^ Likewise, empirical antituberculosis treatment without microbiological confirmation, observed in 9.3% of our patients, is particularly concerning and has been previously described in Brazilian series conducted in tuberculosis-endemic settings where the differential diagnosis may be insufficiently broad.^15^ The two patients initially diagnosed with primary ciliary dyskinesia highlight the phenotypic overlap between these rare diseases, both presenting with chronic sinopulmonary disease, bronchiectasis, and infertility; accurate genetic diagnosis is critical for therapeutic stratification.

Limited access to pediatric and adult pulmonologists also represents a significant barrier to timely diagnosis. This scenario is particularly critical in healthcare systems with unequal distribution of specialized resources, where referral to CF centers remains limited.^11^

From a clinical perspective, cystic fibrosis may mimic more prevalent respiratory disorders, such as asthma and chronic bronchitis, often leading to alternative diagnoses and delays in appropriate investigation. Limited access to essential complementary investigations, particularly chest CT for the detection of bronchiectasis, combined with limited awareness or underutilization of the sweat chloride testing as part of the diagnostic workup further contributes to underdiagnosis.

The median FEV_1_ at diagnosis was 62% of the predicted value (IQR, 49.5-78.0%), indicating that a substantial proportion of patients already had moderate-to-severe airflow obstruction at the time of diagnosis. This is a clinical landmark of considerable prognostic significance: one of the strongest predictors of long-term outcomes in CF is FEV_1_, and patients entering specialized care with established airflow obstruction and bronchiectasis experience worse long-term pulmonary outcomes despite optimal management.^3^ ^,^ ^10^ Furthermore, positive bacterial airway cultures was documented in 84.6% of patients, including mucoid *P. aeruginosa* in 33.3%, indicating advanced microbial airway colonization that is extremely difficult to eradicate once established, further compounding the pulmonary prognosis of late-diagnosed individuals.^8^

Another important factor is the geographic centralization of CF referral centers and diagnostic resources in large urban areas, creating logistical barriers for patients living in peripheral or rural regions and exacerbating health inequities. Additionally, the high degree of population admixture, as observed in countries such as Brazil, is associated with greater CFTR genetic heterogeneity, including the presence of rare variants, often with residual function. These variants contribute to atypical or milder phenotypes, making clinical recognition more challenging than in populations where classic CF mutations predominate, such as in European populations.^6^

The present study should be interpreted within the context of its limitations. First, the present study had a retrospective, two-center cross-sectional design and was conducted at two specialized referral centers, possibly causing selection bias resulting in overrepresentation of patients with more severe or diagnostically challenging disease. Second, the relatively small sample size (n = 54), although adequate for descriptive purposes, limited the statistical power for multivariate analyses and subgroup analyses. Third, spirometry data were available for only 42.6% of patients, precluding a complete lung function analysis of the cohort. Fourth, information regarding symptom onset and prior misdiagnosis relied on retrospective patient and caregiver recall and is therefore subject to recall bias. Finally, longitudinal follow-up data were unavailable, preventing assessment of outcomes after diagnosis.

Despite these limitations, our findings have important implications for both public health and clinical practice in Rio de Janeiro and Brazil. First, they provide direct, locally-generated evidence for the urgent need to strengthen CF NBS programs in the state of Rio de Janeiro through improvements in IRT-based screening algorithms and expansion of confirmatory diagnostic infrastructure, including the availability of sweat chloride testing in facilities and certified CF laboratories. Second, they highlight the need for systematic CF education among primary care physicians, pediatricians, pulmonologists, and emergency physicians, particularly regarding atypical presentations and common diagnostic pitfalls. Finally, in the emerging era of CFTR modulators-drugs that directly target the underlying molecular defect and have transformed CF prognosis in eligible patients-reducing diagnostic delay acquires even greater urgency, as therapeutic benefit appears to be maximal when these agents are initiated before irreversible structural lung damage occurs.^11^ ^,^ ^16^

The implementation of genomic newborn screening and the integration of CF into expanded NBS panels using dried blood spot, next-generation sequencing represent potential future strategies to close the current screening gap in Brazil, particularly for patients with non-classical or mild CF phenotypes that may escape detection by IRT-based protocols.^17^ Until such programs become broadly available, heightened clinical vigilance and a low threshold for sweat chloride testing in patients with recurrent respiratory infections, bronchiectasis, failure to thrive, or otherwise unexplained gastrointestinal manifestations remain the most practical strategy for reducing the unacceptable burden of late CF diagnosis in our population.

This cohort of CF patients diagnosed outside NBS in Rio de Janeiro reveals a critical and largely preventable public health challenge. With 98.1% of patients not identified by NBS, a median age at diagnosis of 11 years, a progressive increase in bronchiectasis with advancing age at diagnosis, and CFRD occurring exclusively among adults, our findings provide compelling evidence that diagnostic delay in CF translates directly into irreversible structural and metabolic harm. The high frequency of prior misdiagnosis (83.3%)-particularly asthma and tuberculosis-further underscores the need to improve clinician awareness and NBS performance. Closing the diagnostic gap in cystic fibrosis in Rio de Janeiro requires urgent, coordinated action at the levels of public health policy, laboratory infrastructure, and clinical education-particularly as highly effective CFTR modulators offer their greatest benefit when the disease is diagnosed and treated early.
